# Translating biomarker insights into practice: a path forward in TA-TMA management

**DOI:** 10.3389/fmed.2025.1550365

**Published:** 2025-05-08

**Authors:** Sonata Jodele, Eleni Gavriilaki

**Affiliations:** ^1^Division of Bone Marrow Transplantation and Immune Deficiency, Department of Pediatrics, Cincinnati Children’s Hospital Medical Center, Cancer and Blood Disease Institute, University of Cincinnati College of Medicine, Cincinnati, OH, United States; ^2^2nd Propedeutic Department of Internal Medicine, Aristotle University of Thessaloniki, Thessaloniki, Greece

**Keywords:** transplant-associated thrombotic microangiopathy, biomarkers, complement, eculizumab, ravulizumab, narsoplimab, pegcetacoplan, iptacopan

## Abstract

Recent advances in the management of transplant-associated thrombotic microangiopathy (TA-TMA) include the harmonization of diagnostic criteria and the identification of high-risk disease features. Individual hematologic and complement biomarkers show moderate specificity when used alone in the detection of TA-TMA in hematopoietic stem cell transplant (HSCT) recipients, but the identification of endothelial injury due to microangiopathic process can be enhanced using longitudinal monitoring of biomarkers and clinical features. An increase in the sC5b-9 level reflects terminal complement activation, a hallmark of TA-TMA pathogenesis that guides therapeutic interventions. In addition, distinguishing physiologic from pathologic complement activation is essential for timely diagnosis of the disease and selection of targeted interventions. Eculizumab therapy, a biomarker-guided C5 blocker, significantly improves clinical outcomes in severe TA-TMA; however, there is a lack of knowledge on how to select second-line complement inhibitors or combination therapies for cases with a suboptimal response to eculizumab. This article proposes practical approaches to increasing the specificity and attributability of TA-TMA diagnostic biomarkers by integrating clinically available supportive diagnostic tests and provides insights into potential biomarkers for currently available novel complement inhibitors. These findings help ensure timely diagnosis, prevent irreversible organ injury, and improve outcomes in HSCT recipients with TA-TMA.

## Introduction

Recently, transplant-associated thrombotic microangiopathy (TA-TMA) has become a well-recognized complication in hematopoietic stem cell transplantation (HSCT). This condition is classified as one of the endothelial injury syndromes that occurs during the early post-transplant period (most commonly within the first 100 days). TA-TMA significantly affects patient survival and long-term outcomes. In the past decade, there have been significant advances in understanding the pathogenesis of TA-TMA, especially regarding complement-mediated endothelial injury. These advances have laid the groundwork for stratifying disease risk and selecting targeted therapies (1). In addition, an international expert group has worked on the harmonization of TA-TMA diagnostic criteria and the associated organ injuries, which is an important step in improving our knowledge about TA-TMA. The expert group reviewed the literature, generated consensus statements regarding the diagnostic and prognostic features of TA-TMA using the Delphi method, and identified future directions with the aim of providing guidance and more uniform approaches to planning prospective studies, data registries, and clinical practice across international settings. They proposed a combination of high-risk biomarkers and clinical features to identify patients at risk of poor outcomes, who may benefit from timely targeted interventions (2). Furthermore, collaboration efforts have led to the development of a pragmatic screening algorithm that is useful for clinical applications.

Despite these efforts, real-life application of TA-TMA biomarkers is challenging as hematologists and transplanters recognize them as non-specific to TA-TMA, especially in patients with concomitant post-HSCT endothelial injury syndromes, such as graft-versus-host disease (GVHD) and sinusoidal obstruction syndrome/hepatic veno-occlusive disease. In addition, in this era of precision medicine, more data are needed regarding the use of the available biomarkers in selecting the appropriate therapeutic agent for each patient (3). The present study offers perspectives on combined expertise and viewpoints on specific areas of interest, current advances, and future directions. These perspectives are supported by a targeted literature search on PubMed for diagnostic biomarkers of TA-TMA included in the harmonization criteria and complement biomarkers that are relevant to the currently available complement blocking agents under investigation.

## PART I: using routine diagnostic biomarkers for TA-TMA diagnosis confirmation and risk assessment

Individual hematologic and complement biomarkers show moderate specificity when used alone in the diagnosis of TA-TMA in HSCT recipients. However, their specificity can be significantly enhanced if they are combined with other supportive tests in the context of microangiopathy and endothelial injury.

### The role of s C5b-9 and rUPCR as diagnostic and prognostic biomarkers

Terminal complement activation measured by increased plasma-soluble C5b-9 (sC5b-9) levels has been identified and investigated as a diagnostic and prognostic biomarker for TA-TMA (4). sC5b-9 is a cornerstone biomarker that is responsible for endothelial injury in the complement pathway, the best-studied pathogenetic pathway in TA-TMA where successful targeted interventions can be made (5).

The complement system is part of our innate immune system that responds to infection, inflammation, and tissue injury, which occur often during the transplantation process. Endothelial injury biomarkers and clinical presentation might be helpful in the timely diagnosis of TA-TMA and risk attribution, in combination with the recognition of normal versus pathologic complement activation (6).

A physiologic increase in sC5b-9 levels is usually mild and transient and occurs in response to the identified triggers such as infections. It tends to be brief as sC5b-9 returns to the normal level with treatment or by controlling the underlying cause. Furthermore, a physiologic increase in sC5b-9 levels does not result in organ damage and is not associated with hematologic or microangiopathic biomarkers, such as consumptive thrombocytopenia and schistocytosis (7).

A pathologic increase in sC5b-9 levels is usually prolonged and is correlated with the concurrent evolution of microangiopathy symptoms. In TA-TMA, the pathologic increase in sC5b-9 levels persists and results in multiorgan dysfunction due to endothelial injury and microthrombosis. Symptoms usually worsen over time without targeted interventions (4). The clinical response of TA-TMA to complement blocking therapy with the C5 inhibitor eculizumab also supports that the increase in sC5b-9 levels is pathologic and is related to complement-mediated endothelial injury (8).

Correct interpretation of whether the increase in sC5b-9 levels is relative or absolute is also extremely important. Physiologically relevant sC5b-9 levels had not been defined in HSCT populations when this biomarker was adopted into clinical practice. Prospective screening studies on children and young adults determined that the pretransplant median value of sC5b-9 is 92 ng/mL (range 47–127 ng/mL) and the increased level (abnormal) of sC5b-9 according to current laboratory values is ≥244 ng/mL. This study showed that the doubling of the pretransplant sC5b-9 level, or sustained activation of sC5b-9 for more than 2 weeks, even if it remained “within normal limits” by the laboratory-determined norms, signifies clinically relevant terminal complement activation associated with an increased risk of multiorgan dysfunction and death (4). A “normal” sC5b-9 laboratory level should not preclude the consideration of complement blocking therapy in HSCT recipients with other diagnostic biomarkers for TA-TMA or with concurrent relevant organ injury, especially if the pretransplant baseline sC5b-9 level is unknown or sC5b-9 results are unavailable. Research on the pharmacokinetics of eculizumab in TA-TMA indicates that those with normal sC5b-9 levels show faster response to treatment and require lower doses for therapy as they have fewer ligands available for binding (9). In addition, monitoring the eculizumab drug level in patients with normal sC5b-9 levels provides further clinical evidence of localized complement activation in the affected organ rather than a systemic response, if the drug is being “consumed” based on drug trough levels (10). However, other endothelial injury syndromes that often precede TA-TMA, such as GVHD, may be associated with slight increases in sC5b-9 levels, as reported in adults (11).

Thus, monitoring interpersonal sC5b-9 levels allows us to longitudinally distinguish between physiologic and pathologic activation. Doubling of baseline sC5b-9 levels or a sustained increase in sC5b-9 levels can provide diagnostic reassurance when used in conjunction with other TA-TMA biomarkers and guide timely treatment. In addition to being one of the diagnostic biomarkers, sC5b-9 signifies a high risk of organ injury and an increased risk of mortality, which has been shown by multivariate analysis in retrospective and prospective screening studies (4).

Proteinuria, which is identified by a random urine protein creatine ratio (rUPCR) of ≥1 mg/mg, has been identified as a hallmark of TA-TMA-associated kidney injury in pediatric and young adult HSCT recipients. Previous studies used an rUPCR of ≥2 mg/mg as a high-risk TA-TMA feature, which was later reduced to an rUPCR of ≥1 mg/mg to prevent irreversible kidney injury based on prospective screening and TA-TMA survivor monitoring (2, 12). While low-degree proteinuria (rUPCR = 0.2–0.99 mg/mg) can be identified in HSCT recipients with significant nephrotoxic effects from chemotherapy, medications, or infections, high-degree proteinuria of an rUPCR of ≥1 mg/mg is observed nearly exclusively in patients with TA-TMA (13). An rUPCR value of ≥1 mg/mg, if evaluated longitudinally starting with the pretransplant baseline value and in the context of other microangiopathic changes, serves as an indicator of kidney injury due to TA-TMA. Additional tests can increase the specificity of proteinuria attribution to glomerular endothelial injury. A higher-than-normal albumin/creatine ratio can indicate glomerular injury. Microalbuminuria may confirm early-stage glomerular injury as it usually precedes overt proteinuria. These additional glomerular injury biomarkers can support renal involvement in TA-TMA, especially in combination with an abnormal or increasing rUPCR. Cystatin C GFR is a preferred glomerular filtration (GFR) biomarker in children, especially in those with abnormal muscle mass (14). It has been successfully applied in the longitudinal monitoring of children with TA-TMA. All these glomerular injury biomarkers are readily available for clinical use and are inexpensive (Table 1). In addition, urokinase plasminogen activator receptor (uPAR) has emerged as another potential AKI biomarker in TA-TMA. It plays a key role in the plasminogen activation system and may indirectly reflect complement-mediated renal injury; however, further studies should be carried out before incorporating uPAR into clinical practice (15).

**Table 1:** TA-TMA diagnostics and supportive tests.

A. TA-TMA harmonization panel consensus–recommended Diagnostic criteria, modified Jodele criteria		B. Supportive tests aiding biomarker Attribution to TA-TMA
(1) Biopsy-proven disease (kidney or GI)		Affected tissue histologic diagnosis (any affected organ)
(2) Clinical criteria: must meet ≥4/7 criteria within 14 days at two consecutive time points		ADAMTS13 activity to rule out TTP
Anemia*	Defined as one of the following: Failure to achieve transfusion independence for pRBCs despite evidence of neutrophil engraftment Hemoglobin decline from patient’s baseline by 1 gm/dL New onset of transfusion dependence Rule out other causes as the sole cause of anemia such as AIHA and PRCA	Haptoglobin. Haptoglobin degradation products Reticulocytes Free plasma hemoglobin to confirm hemolysis Direct Coombs
Thrombocytopenia*	Defined as one of the following: Failure to achieve platelet engraftment Higher-than-expected platelet transfusion needs Refractoriness to platelet transfusions 50% reduction in baseline platelet count after full platelet engraftment	Antiplatelet antibodies, when applicable Disseminated intravascular coagulation panel
Increased LDH	Above the upper limit of normal for age	Free plasma hemoglobin indicates intravascular hemolysis Increased reticulocyte count can be observed in response to hemolysis Increased indirect bilirubin supports the diagnosis of hemolysis when associated with LDH Urinary hemoglobin indicates breakdown of RBCs in association with increased LDH
Schistocytes	Present	Manual review of peripheral blood smear Tissue evidence of extravasated schistocytes
Hypertension	>99th percentile for age (<18 years) or systolic BP ≥ 140 or diastolic BP ≥ 90 (≥18 years)	Change in the number of antihypertensive medications required for hypertension control Rapid onset correlated with microangiopathic hemolysis, proteinuria, and complement activation Severe manifestation leading to end-organ damage Lack of response to standard therapy (diuretics, calcium channel blockers) Response to complement blocking agents
Increased plasma sC5b-9	Higher than or equal to the upper limit of normal	Differentiation between physiologic and pathologic sC5b-9 increase **Physiologic** Mild and transient, most commonly as response infection Return to baseline with trigger resolution Lack of severe hemolysis or microangiopathy Lack of organ dysfunction **Pathologic** Significant and sustained increase, typically doubling from the baseline Associated with microangiopathy leading to endothelial injury Results in organ dysfunction Prolonged and worsening symptoms Response to complement blockade
	sC5b-9 doubled from pre-HSCT baseline and sustained increase in sC5b-9	Persistently increased sC5b-9 levels on serial measurements, particularly when doubled from the baseline or sustained for over 2 weeks, are associated with severe TA-TMA and multiorgan dysfunction
	Response to C5 inhibitors	Reduction in sC5b-9 levels using C5 inhibitors confirms the role of clinically meaningful complement activation in endothelial injury in TA-TMA
Proteinuria	≥1 mg/mg rUPCR	Cystatin C GFR Increased serum creatinine (normal serum creatinine does not rule out AKI in pediatric HSCT recipients) Urine microalbumin Urine albumin/creatinine ratio (>30 mg/g) uPAR#

pRBCs, packed red blood cells; AIHA, autoimmune hemolytic anemia; PRCA, pure red cell aplasia; BP, blood pressure, GI, gastrointestinal, rUPCR random urine protein/creatinine ratio, TTP, thrombotic thrombocytopenic purpura, GFR, glomerular filtration rate; uPAR, urokinase plasminogen activator receptor.

*Indicates clarification from published Jodele et al. criteria.

^#Not used clinically at this time.^

### Hypertension due to microangiopathic process

Hypertension caused by TA-TMA occurs due to primary and direct renal endothelial injury and microthrombosis, which activates the renin–angiotensin–aldosterone system. It can lead to the vasoconstriction of the affected renal arterioles, resulting in severe or intractable hypertension. Some key features that help attribute hypertension to TA-TMA can be defined as the “TA-TMA hypertension triad”: (1) rapid onset, which can be severe and/or persistent, (2) association with other markers of microangiopathy, and (3) evidence of end-organ damage. A rapid and acute increase in blood pressure with concurrent signs of other microangiopathy symptoms, such as increased sC5b-9 levels and/or concurrent proteinuria indicated by the rUPCR, supports the diagnosis of hypertension due to TA-TMA. Severe and sustained hypertension often results in organ damage, neurologic complications Posterior Reversible Encephalopathy Syndrome (PRES), and cardiac and renal injury. Hypertension due to TA-TMA is often resistant to standard antihypertensive therapy with calcium channel blockers and diuretics. It often requires multidrug therapy (>2 medications, not including diuretics) that is more than that administered for hypertension-inducing conditions after HSCT, such as Calcineurin inhibitor (CNI) and steroid use, and in those with pre-existing hypertension, a significantly improved medical therapy compared with their baseline. The response of hypertension to complement blocking therapy serves as another factor supporting the role of TA-TMA in hypertension. In children undergoing HSCT, personalized blood pressure thresholds should be used to monitor hypertension, accounting for age, sex, and height (16–18).

### Practical approaches to overcoming the limitations of hematologic tests in TA-TMA diagnosis

Prospective TA-TMA screening is the most relevant clinical strategy that can be used to understand the role of hematologic markers in TA-TMA, especially in the pre-engraftment and early post-transplant period, as analyzed in detail by Dandoy et al. (19). A crucial aspect in this evaluation is organ injury assessment, with the gastrointestinal (GI) assessment being exceedingly difficult in the presence of concurrent GVHD. Interestingly, transplanters and pathologists need to consider the suggested entity of intestinal TMA as well in the case of an isolated GI TMA without prominent systemic TMA (6, 11, 20). Furthermore, GI bleeding has to be evaluated in the context of other TMA biomarkers.

Since TA-TMA diagnostic criteria include only a few hematologic biomarkers, medical care providers should use other available diagnostic tests as appropriate to support the role of biomarkers in TA-TMA (Table 1). While increased LDH levels, schistocytes, anemia, and thrombocytopenia can be observed in various conditions, the simultaneous appearance of these markers during prospective monitoring is highly indicative of TA-TMA. In TA-TMA, thrombocytopenia and anemia are consumptive; therefore, transfusion dependence and intensity are more indicative of a microangiopathic process than a low cell number.

Hemolytic anemia in those who underwent transplantation can be differentiated from other causes of anemia using supportive tests such as Lactate Dehydrogenase (LDH), plasma-free hemoglobin levels, indirect bilirubin levels, and reticulocyte count. Although a low haptoglobin level was historically included as one of the hemolytic anemia biomarkers in diseases such as atypical hemolytic uremic syndrome (aHUS), it is not uncommon to observe increased haptoglobin levels in TA-TMA diagnosis (12). An increased haptoglobin level does not rule out TA-TMA as it indicates ongoing systemic inflammation as an acute-phase response along with hemolysis and is associated with poor outcomes in TA-TMA. Furthermore, increased haptoglobin levels can aid in assessing disease severity if interpreted along with other high-risk disease biomarkers such as sC5b-9. Moreover, the haptoglobin degradation product can provide further useful information in assessing hemolysis, but it has not yet been available as a clinical test (21).

Some coagulation and thrombosis biomarkers can support the diagnosis of TA-TMA. D-dimer can have both diagnostic and prognostic significance if it is evaluated in the clinical context as it is a sensitive marker of fibrinolysis and thrombosis, but not specific to TA-TMA (22). Increased D-dimer levels and thrombocytopenia may be suggestive of microangiopathy when they occur together; however, caution is required in patients with suspected Disseminated intravascular coagulation (DIC). Nevertheless, a normal D-dimer level does not rule out TA-TMA as the D-dimer level may not be increased if thrombi are not actively lysed or the fibrin degradation rate is slow.

Thrombomodulin is a natural anticoagulant and a regulator of complement system activation. In addition, it protects the endothelium from thrombus-mediated and complement-mediated injury. Thrombomodulin is released from damaged endothelial cells while also being a part of the complement system; thus, thrombomodulin levels can be increased in TA-TMA (6, 11). The levels of thrombin–antithrombin complex (TAT) may increase as a result of platelet activation and microthrombosis. Fibrinogen levels may be decreased in TA-TMA as fibrinogen is consumed during thrombus formation. Fibrinogen degradation products are produced during fibrinolysis of fibrin, whose levels can be increased in TA-TMA due to the breakdown of microthrombi (23).

A reduced activity of ADAMTS13 (a disintegrin and metalloproteinase with a thrombospondin type 1 motif, member 13) may be observed with hemolysis in TA-TMA due to platelet consumption and the aggregation of larger von Willebrand factor multimers with microthrombosis, but this usually remains >10% (24). An ADAMTS13 activity test should be recommended, but targeted therapy for TA-TMA should not be delayed while waiting for the results. A reduced activity of ADAMTS13 can be attributable to the release of ultra-large von Willebrand factors in microthrombus formation.

### Defining high-risk disease and avoiding Bias in clinical outcome assessment

TA-TMA risk stratification is essential not only for guiding clinical care but also for the assessment of clinical study outcomes. It is not uncommon that clinicians interchangeably compare overall TA-TMA outcomes and high-risk TA-TMA outcomes, thus introducing significant bias in evaluating therapy response and strategizing therapeutic studies. It has been well documented that high-risk TA-TMA, if untreated timely, results in dismal outcomes with a 1-year post-transplant survival of <20% and an overall survival of <10%; however, overall survival in all patients with TA-TMA (combining patients with mild, moderate, and severe disease) is approximately 50% (12). This is influenced by low-risk or purely hematologic TA-TMA, which does not result in organ injury and does not affect survival without any additional interventions. Although the most powerful data are generated by randomized clinical studies, randomization of HSCT recipients with high-risk TA-TMA early after transplant is unethical as TA-TMA can progress to a lethal condition in a matter of days even though there is proven benefit for early intervention with complement blocking therapy in this population. The first prospective multi-institutional eculizumab trials on children and young adults investigated early intervention with eculizumab in patients with TA-TMA and multiorgan endothelial injury syndrome. This study was conducted under Investigational New Drug Application (IND) number 136738 and registered at www.clinicaltrials.gov as #NCT03518203. All subjects in this study had to meet the high-risk disease criteria: increased sC5b-9 levels, an rUPCR of >1 mg/mg, and having multiorgan injury at the beginning of the therapy. Eculizumab therapy was initiated at a median of 2 days (IQR 2–4) after high-risk TMA diagnosis. Achieving 71% survival 6 months after TA-TMA diagnosis is a significant improvement compared with that in historical controls with the same risk (18% survival). However, outcomes of high-risk TA-TMA studies, especially those supported by high-risk biomarker monitoring, cannot be directly compared with studies that do not risk-stratify patients at enrolment (8).

Recent TA-TMA consensus definitions require only one high-risk feature to consider initiating therapy to prevent irreversible organ injury. Numerous reports have proposed that high sC5b-9 levels, rUPCR >1 mg/mg, and multiorgan dysfunction are risk factors for TA-TMA (2). Concurrent grade II–IV GVHD has been shown to be involved in the development of TA-TMA (25). Furthermore, unsupported theories still exist that treating GVHD alone can resolve TA-TMA and that by eliminating GVHD, TA-TMA can also be eliminated. Recent findings from studies using post-transplant cyclophosphamide indicate that eradicating GVHD will not necessarily eradicate TA-TMA (26). Additional evidence is emerging that ST2, which is currently considered one of the predictors for steroid-refractory GVHD, is in fact an endothelial injury marker signifying TA-TMA (27). Further studies are ongoing in children and adults to specifically address these questions.

Infections are considered a high-risk feature when present in conjunction with other microangiopathy markers, especially a sustained increase in sC5b-9 levels. This indicates that physiologic complement activation in response to infection is not aborted by antimicrobial therapy and persists to pose a risk of endothelial injury.

LDH x2 UNL has been included in harmonization definitions as a high-risk feature based on a few retrospective studies that have not assessed complement activation and/or proteinuria by rUPCR (28, 43). Literature from transplant centers prospectively screening for TA-TMA does not support the increase in LDH levels as a high-risk feature, at least in the pediatric population (19).

Finally, it is important to highlight the potential role of genetic biomarkers that needs to be further explored in future studies. Genetic variants in complement-related genes have been identified in pretransplant samples of both pediatric and adult patients who developed TA-TMA (6, 10, 11). Nevertheless, there are critical limitations that hinder the potential use of these genes in clinical practice, one of the primary limitations being the unknown clinical relevance of many variants or the combination of them.

## PART II: biomarkers and therapeutic strategies for optimizing complement blockade in TA-TMA

C5 blocker eculizumab is the first-in-class complement inhibitor that has been approved for paroxysmal nocturnal hemoglobinuria (PNH), aHUS, myasthenia gravis, and neuromyelitis optica. Currently, there are no approved complement-blocking agents for TA-TMA. Eculizumab is the first complement blocker to be used as an off-label drug in HSCT recipients with TA-TMA. It inhibits C5 cleavage into C5a and C5b, thus preventing the formation of C5b-9 and reducing endothelial injury (29).

The safety and efficacy of eculizumab have been shown in both adult and pediatric patients with TA-TMA (44, https://doi.org/10.1016/j.tru.2024.100186). Jodel et al. have revealed that among six patients with TA-TMA who were treated with eculizumab, four patients showed resolution of the disease (12, PMID: 24370861). Fontbrune et al. have assessed the outcomes of eculizumab therapy in 12 adult and pediatric patients with TA-TMA and shown that the hematologic response of the patients was 50% (45, PMID: 25651309). Similarly, Rudoni et al. have reported a hematologic response of 70% in their study population of 10 TA-TMA allogeneic-HSCT recipients (46, PMID: 29920784). Furthermore, Benítez Carabante et al. have included 29 pediatric patients in their study and reported that 19 patients (65.5%) responded to eculizumab, of whom 17 (58.6%) achieved complete response and 2 (6.9%) achieved partial response (47, PMID: 38521410). It is worth highlighting that the differences between the reported outcomes in these published studies might be attributable to the different response criteria used. In addition, multicenter collaboration, prospective study design, and development of harmonized response criteria are crucial to better understand the factors associated with the response to eculizumab therapy for TA-TMA.

Extensive knowledge has been gained from biomarker-guided pharmacokinetics studies on eculizumab in HSCT recipients with TA-TMA (9, 10, 30, 31). Monitoring of the sC5b-9 level and eculizumab drug level has been successfully integrated into clinical practice as precision therapy, resulting in significantly improved outcomes. The decrease in the sC5b-9 level to the normal level following C5 blockade indicates the effective inhibition of the terminal complement pathway. If the sC5b-9 level remains increased even after eculizumab therapy, the most common reason will be an inadequate eculizumab dosing regimen.

The therapeutic trough level (the lowest concentration before the next dose) of eculizumab is ≥100 μg/mL for HSCT recipients with TA-TMA. A lower drug level may result in incomplete complement blockade and disease progression. Eculizumab drug level *per se* can serve dual purposes: to assess adequate drug dosing during therapy and to determine when therapy can be discontinued as it is a ligand-based therapy and the drug is cleared rapidly with active disease (as there are a high number of ligand to bind) and accumulated in blood when complement overactivation is resolved (no ligand to bind) (9). However, eculizumab requires dosing every 2 weeks, a limitation that has been overcome by ravulizumab, which requires less frequent dosing and has been approved for eculizumab’s indications (29). However, direct clinical evidence for ravulizumab in TA-TMA is still lacking as clinical studies on ravulizumab are ongoing.

Furthermore, the CH50 level can be used as a surrogate biomarker for therapeutic eculizumab drug level. CH50 is a functional assay that measures the overall activity of the classical complement pathway and is influenced by C5 cleavage. If eculizumab effectively inhibits C5, then CH50 levels will be suppressed below the lower normal limit as C5b-9 formation is prevented (31). The same should be applicable to ravulizumab, but this has not yet been confirmed in clinical practice. Recent clinical and translational studies have shown that all complement pathways, classic, lectin, alternative, and terminal, can be involved in the pathogenesis of TA-TMA, offering additional options for therapy choices, but relevant biomarkers for the representative drugs have not yet been studied (32). Ultimately, sC5b-9 is the most suitable biomarker to assess the effectiveness of any complement blocker as it directly reflects complement activation on the endothelium, which is central in TA-TMA pathogenesis. Despite this, with several novel complement blockers entering the clinical arena, we strive to guide drug selection by scientific evidence, not just by drug availability. Beyond CH50, novel functional assays have been introduced for a more reliable evaluation of complement function (33, 34). However, these assays have not been studied in TA-TMA and their clinical applicability remains limited to date.

Information on biomarkers with the potential for guiding therapy indication and response assessment for currently available specific complement blockers is presented in Table 2. Understanding the role of these complement blockers is essential to guide second-line therapy in TA-TMA cases with suboptimal response to eculizumab. The majority of these biomarkers are available as clinical tests and can be further studied for applicability.

**Table 2:** Complement blocking agents studied for thrombotic microangiopathies and relevant biomarkers.

Complement target	Targeting agent	Biomarker to determine therapeutic indication or effect
MASP2	Narsoplimab	C3d, C4d, C4 cleavage products, sC5b-9, and MBL (indirect)
C3	Pegcetacoplan	C3a, C5a, C3b, C3d, sC5b-9, AH50, sC5b-9, Wieslab, complement alternative pathway, and mHam2.0
C5	Eculizumab	sC5b-9, C5a, and mHam2.0
	Ravulizumab	CH50 as the surrogate marker for eculizumab drug level Upstream markers C3a and C4d can also be used if sC5b-9 is not available
Factor B	Iptacopan	Factor B, Bb, C3a, C5a C3bBbP, sC5b-9, Wieslab, complement alternative pathway, and mHam2.0

MASP2, mannan-binding lectin serine protease 2; CH50, total complement activity; AH50, alternative hemolytic activity; mHam, modified Ham test; sC5b-9, soluble C5b9; MBL, mannose-binding lectin.

Lectin pathway activation plays a significant role in coagulation activation and microthrombosis. If eculizumab fails in TA-TMA due to overactivated coagulation, for example in patients with intestinal bleeding, who exhibit fast eculizumab clearance, the mannan-binding lectin serine protease 2 (MASP2) inhibitor narsoplimab can be an appropriate therapy (35, 36). MASP-2 activity, C3d, C4d, C4 cleavage products, and ultimately sC5b-9 level can indicate lectin pathway activation. Mannose-binding lectin (MBL) is another key component of the lectin pathway. MBL tests are clinically available, so an increased MBL level can serve as a marker of lectin pathway activation (37, 38). Although narsoplimab does not directly modulate MBL, it can be informative when used in conjunction with other biomarkers. In their single-arm study on 28 TA-TMA adult patients treated with narsoplimab, Khaled et al. found a response rate of 61%, which was evaluated based on laboratory TA-TMA markers, organ function improvement, and freedom from transfusion (48, PMID: 35439028). Castelli et al. have reported similar response rates to narsoplimab (65%) in their real-world study, in which 20 adult and pediatric patients were included (35). Moreover, several real-world case reports and case series have indicated the safety and efficacy of narsoplimab in both adult and pediatric TA-TMA patients (39, 40). Mechanistically, blocking of the lectin pathway by narsoplimab might be beneficial due to not only the association between the lectin pathway and coagulation but also the potential involvement of the lectin pathway in TA-TMA (32). Recent findings have confirmed the efficacy of narsoplimab in severe refractory TA-TMA (36).

Even if eculizumab does not adequately inhibit C5 cleavage, sC5b-9 will still be formed and C5a levels remain increased. This could be attributable to inadequate drug dosing. In patients who receive eculizumab, the detection of C5a shows that C5 cleavage occurs, indicating insufficient drug efficacy. When eculizumab is administered and the patient shows no clinical improvements (ongoing hemolysis or organ dysfunction), it may suggest the dysregulation of an alternative pathway upstream to the terminal complement pathway. Several studies support the idea that in TA-TMA, alternative pathway blockade may be required due to increased Ba levels (28, 41, 42). Factor B inhibitors such as iptacopan might be considered for blocking the alternative pathway. Iptacopan was approved as an oral monotherapy for PNH in 2023 and is currently being studied for aHUS. Monitoring factor B levels and Bb fragment in plasma, in addition to C3a, C5a, C3bBbP, and sC5b-9, can help guide the indication to initiate factor B blocker and assess therapy response. From a pharmacokinetics standpoint, factor B inhibitors may be preferred over factor D inhibitors such as danicopan as the levels of the latter increase and fluctuate with renal dysfunction, posing additional challenges in achieving adequate inhibition.

C3 inhibitors (such as pegcetacoplan) potentially provide a broader inhibition of complement activation, addressing both alternative and classical pathway dysregulation in TA-TMA. Pegcetacoplan acts by inhibiting C3 convertase, which blocks both alternative and classical complement pathways, leading to a reduction in the generation of C3a, C5a, and C5b-9. These biomarkers can be used in therapy monitoring. A reduction in AH50 activity after pegcetacoplan administration indicates the effective inhibition of the alternative pathway.

The classical complement pathway, namely C1q, has been implicated as a complement cascade initiator in transcriptome analyses of TA-TMA patients, but the mechanism of C1 blockers in TA-TMA has not yet been studied (32). C3d, C4d, and C1q biomarkers may guide the use of C1s inhibitors such as sutimlimab. Interestingly, a recent study on aHUS has shown that classical pathway activation may contribute to its pathogenesis, additionally suggesting a novel functional assay (modified Ham test, version 2.0, aka mHam2.0) as a model for diagnostic and monitoring purposes (33). Further data regarding the diagnostic role of mHam2.0 in TA-TMA are essential for examining the diagnostic accuracy of this approach.

Combining multiple complement inhibitors can provide a broader therapeutic strategy for patients with an incomplete response to C5 blockers by addressing complement dysregulation at multiple points in the complement cascade, but such approaches need to be systematically studied to address treatment efficacy, side effects, and other factors.

## Discussion

Recent advances in harmonizing TA-TMA diagnostic criteria and risk stratification have aided in guiding clinical practices and strategizing clinical studies. Prospective longitudinal TA-TMA monitoring and integrating clinically available supportive biomarkers enhance the specificity of TA-TMA diagnosis and facilitate the timely application of targeted therapies.

Effective complement-blocking therapies, such as eculizumab, have significantly improved the outcomes, with biomarkers playing a crucial role in both therapy initiation and monitoring. The availability of additional complement blockers targeting different pathways—classical, lectin, alternative, and terminal—expands the therapeutic options for patients with suboptimal responses to first-line treatments. Clinically available biomarkers such as sC5b-9, CH50, AH50, MBL, and C5a, among others show potential for tailoring these therapies and assessing their efficacy. A wider accessibility of these markers and potentially novel functional assays, such as mHam2.0, are highly warranted.

The development of biomarker-guided diagnostic and therapeutic strategies is essential to further improve the outcomes and advance the clinical practice in TA-TMA management. Collaborative efforts to validate the application of biomarkers in clinical trials will further support their integration into personalized treatment paradigms.

## Data Availability

The original contributions presented in the study are included in the article/supplementary material, further inquiries can be directed to the corresponding author.
